# Cytotoxic and Apoptotic Activity of Aglaforbesin Derivative Isolated from *Aglaia loheri* Merr. on HCT116 Human Colorectal Cancer Cells

**DOI:** 10.31557/APJCP.2021.22.1.53

**Published:** 2021-01

**Authors:** Norielyn N. Abalos, Virgilio D. Ebajo Jr., Drexel H. Camacho, Sonia D. Jacinto

**Affiliations:** 1 *Institute of Biology, University of the Philippines Diliman, 1101, Quezon City, Philippines. *; 2 *Department of Biology, University of San Carlos-Talamban Campus, 6000, Cebu City, Philippines. *; 3 *NMR Laboratory, Central Instrumentation Facility, De La Salle University, Laguna Campus, LTI Spine Road, Laguna Boulevard, Barangays Biñan and Malamig, Biñan City, Laguna, Philippines. *; 4 *Chemistry Department, De La Salle University, 2401 Taft Avenue, 0922 Manila, Philippines. *

**Keywords:** Cytotoxicity, MTT Assay, Aglaia loheri, apoptosis, aglaforbesin derivative

## Abstract

**Background:**

The genus *Aglaia* (Meliaceae) is an established source of many anticancer compounds. The study evaluated the leaf extracts of *Aglaia loheri*, a tree native to the Philippines, as potential source of anticancer compounds.

**Methods:**

Using bioassay-guided fractionation, *A. loheri* leaf extract was subjected to various chromatographic techniques and step-wise application of MTT assay on human colorectal carcinoma cells, HCT116, to determine the cytotoxic fractions. The most cytotoxic HPLC isolate was structurally identified using 1D and 2D NMR and its apoptotic effect was assessed by JC-1 staining, caspase 3/7 assay and TUNEL assay.

**Results:**

After stepwise chromatography fractionation, an HPLC isolate, structurally identified as aglaforbesin derivative (AFD), demonstrated potent cytotoxicity against HCT116. AFD exhibited strong toxicity (IC_50_ = 1.13 ±0.07 µg/mL) and high selectivity on HCT116 than normal human kidney cells (HK-2). AFD-induced toxicity to HCT116 is possibly through the stimulation of the apoptotic signaling pathway via caspase 3/7 activation and DNA fragmentation independent of mitochondrial membrane depolarization.

**Conclusion:**

AFD exhibited selective cytotoxicity and apoptotic activity to HCT116 and could be further developed as anticancer drug lead.

## Introduction

Global cancer incidence (18.1 million) and mortality (9.6 million) estimates remain high despite existing interventions, indicating the need to discover new and more effective anticancer agents (Bray et al., 2018). One of the most common types of cancer is colorectal cancer with about 1.1 million new cases and 551, 269 deaths per year (Bray et al., 2018). Safer treatments that directly target cancer cells with little or no harmful side effects on normal cells is the trending research direction in anticancer drug discovery. Plants have been an effective source of these bioactive compounds exerting antitumor functions, including cytotoxicity, induction of apoptosis, antioxidant activity, modulation of cell growth factors, and inhibition of angiogenesis (Singh et al., 2016). The Philippine megadiverse forests potentially harbor plants with anticancer properties waiting to be discovered. One prospective candidate is *Aglaia loheri*, a native species locally known as Balubar. It is part of the diet of the Aetas, an indigenous tribe in the Philippines, who use the plant for medicinal and nutritional purposes (Dapat et al., 2013). Many *Aglaia* species have been used in traditional medicine to treat fever, cough, asthma, inflammations, wounds and tumors (Janaki et al., 1999; Proksch et al., 2005; Ebada et al., 2011). 

Isolation of compounds from different *Aglaia* species show highly cytotoxic to non-cytotoxic activity against an array of human cancer cells. Several novel compounds cytotoxic against different cancer cell lines have been isolated from *Aglaia* species e.g., silvestrol from *Aglaia foveolata* (King et al., 1982), rocagloic acid from *Aglaia elliptifolia* (Wang et al., 2001) and aglaroxin A 1-O-acetate from *Aglaia edulis* (Kim et al., 2006). Previous studies on *A. loheri* leaves showed high to moderate cytotoxicity of crude extract against HCT116 and A549 cells (Canoy et al., 2011). Isolated compounds – pinasterol, trilinolein, and phytyl fatty acid were found to be cytotoxic against HCT116 (Ragasa et al., 2012) and a phenolic ester (Maldi 531.2[M + H]+) exhibiting cytotoxicity against human leukemic cells CCRF-CEM and its multidrug-resistant subline CEM/ADR5000 (Dapat et al., 2013). 

This work is focused on screening for the most active extracts and fractions from *A. loheri* leaves that possess strong cytotoxic activity against human colorectal cancer cell line, HCT116. The study provides initial information on the mode of cell death induced by the most cytotoxic HPLC isolate obtained as well as its chemical identity. Altogether, the results of the study strengthen our understanding on the cytotoxic properties of *A. loheri* extracts and the potential of its cytotoxic isolates to be used as drug lead for future development as therapeutic agent. 

## Materials and Methods

*Plant collection and crude extraction*

Leaves of *A. loheri* were collected from Mt. Lamao, Bataan, Philippines. Identification was verified at the Jose Vera Santos Herbarium of the Institute of Biology, University of the Philippines, Diliman, Quezon City, where voucher specimen (accession number 21418) was deposited. Leaves were washed, air-dried, ground to powder and were macerated in distilled methanol at room temperature for at least 48 hours and filtrate was concentrated using a rotary evaporator at 40°C.

*Bioassay-guided fractionation*

*A. loheri* leaf methanolic extract was sequentially advanced through liquid partitioning, gravity column chromatography (GCC), flash chromatography (FC), size exclusion chromatography (SEC) and reverse-phase high performance chromatography (RP HPLC). Fractions with significant yield were constituted at 10 mg/mL DMSO and screened for toxicity against selected cell lines by 3-(4,5- dimethyl2-thiazolyl)-2,5-diphenyl-2H-tetrazolium bromide (MTT) assay. The most cytotoxic and high yielding fractions were tested against each cancer cell line to determine the concentrations inhibiting viability by 50% (IC_50_). 

The crude extract was partitioned successively with n-hexane, ethyl acetate, and water. The partitions were concentrated using rotary evaporator at 40°C while the aqueous partition was lyophilized. The most active ethyl acetate extract was subjected to GCC eluted with gradient of n-hexane, ethyl acetate and methanol. The fractions were monitored by thin layer chromatography (TLC) and similar eluents were combined. The high yielding, cytotoxic fraction 26 was subjected to FC in Biotage Isolera™ (Biotage, Sweden) using gradient of n-hexane, ethyl acetate and methanol. Similar eluents were pooled, affording 14 fractions. Fraction FC10 demonstrated strong cytotoxicity and was subjected to SEC using Sephadex LH-20, affording 10 fractions. The cytotoxic and high yielding SEC fraction 7 was further purified by semi-preparative HPLC (mobile phase: 70% ACN and 30% H_2_O) using Luna^®^ 5 µm C18 100 Å, 250 mm length, 10 mm internal diameter (Phenomenex Inc., CA, USA). HPLC isolation afforded the cytotoxic HPLC7 fraction (6 mg; *t*_R_=13.5 min).

*NMR analysis*

Nuclear magnetic resonance (NMR) spectra of HPLC7 was recorded on a JEOL ECZR spectrometer at 600 MHz for ^1^H NMR and 150 MHz for ^13^C NMR spectra. Chemical shifts values are given in ppm relative to residual DMSO (^1^H NMR: δ 2.50; ^13^C NMR: δ 39.51) solvent. Coupling constants (*J*) were reported in Hz with the following splitting abbreviations: s = singlet, d = doublet, and m = multiplet.

HPLC7: ^1^H NMR (DMSO-d6, 600 MHz) δ 4.56 (d, *J* = 10.8 Hz, 1H, H-3), 3.61 (d, *J* = 1.8 Hz, 1H, H-4), 6.26 (s, 1H, H-9), 3.93 (s, 1H, H-10), 4.99 (m, 1H, H-13), 1.40 (m, 1H, H-14a), 1.25 (m, 1H, H-14b), 1.8 (m, 2H, H-15), 3.30 (m, 1H, H-16a), 3.15 (m, 1H, H-16b), 2.15 (m, 1H, H-19), 0.85 (d, *J* = 6.6 Hz, 3H, H-20), 0.90 (d, *J* = 6.6 Hz, 3H, H-21), 8.00 (m, 2H, H-2’/H-6’), 6.95 (m, 2H, H-3’/H-5’), 7.2 (m, 2H, H-2”/H-6”), 6.97 (m, 2H, H-3”/H-5”), 6.84 (m, 1H, H-4”), 3.78 (s, 3H, OCH_3_-6), 3.66 (s, 3H, OCH_3_-4’), 5.92 (d, *J* = 1.8 Hz, 1H, OCH_2_O-a), 6.18 (d, *J* = 1.8 Hz, 1H, OCH_2_O-b). ^13^C NMR (DMSO-d6, 150 MHz) δ 148.0 (C-1a), 86.5 (C-2), 62.7 (C-3), 62.5 (C-4), 82.1 (C-5), 118.9 (C-5a), 141.9 (C-6), 131.1 (C-7), 152.2 (C-8), 93.5 (C-9), 85.9 (C-10), 170.5 (C-11), 62.8 (C-13), 21.7 (C-14), 34.9 (C-15), 45.0 (C-16), 174.6 (C-18), 36.7 (C-19), 18.8, (C-20), 19.7 (C-21), 131.0 (C-1’), 129.8 (C-2’/C-6’), 112.7 (C-3’/C-5’), 160.1 (C-4’), 142.0 (C-1”), 130.8 (C-2”/C-6”), 129.2 (C-3”/C-5”), 127.3 (C-4”), 58.0 (OCH_3_ – 6), 55.5 (OCH_3_ – 4’), 105.4 (O-CH_2_-O).

*Cell culture*

The HCT116 and normal human kidney proximal tubule epithelial cells (HK-2), were obtained from the American Type Culture Collection (ATCC, VA, USA). HCT116 was grown in McCoy’s 5a medium (Gibco, NY, USA) supplemented with 10% fetal bovine serum (FBS) (Gibco, NY, USA), 1.5% NaHCO_3_, 1% penicillin/streptomycin (100 U/mL) (Gibco, NY, USA) and 1 µg/mL gentamicin (Gibco, NY, USA). HK-2 was grown in Dulbecco’s modified Eagle’s medium: nutrient mixture F-12 (DMEM/F12) (Gibco, NY, USA) supplemented with 10% FBS, 1% penicillin/streptomycin (100 U/mL) and gentamicin (1 µg/mL). Cells were maintained in T25 culture flasks at 37°C, 5% CO_2_ and 95% humidity. When cells reached 80-90% confluence, they were harvested using 0.05% trypsin solution (Gibco, NY, USA). 

*Assessment of cytotoxicity using MTT assay*

Cytotoxicity of *A. loheri* against HCT116 was determined using MTT assay, a colorimetric test to measure the reduction of MTT by mitochondrial dehydrogenase to purple formazan crystals in live cells. The National Cancer Institute (NCI) established that extracts with IC_50_ values <30 µg/mL against cancer cell lines are promising for isolation of bioactive compounds (Suffness and Pezzuto, 1990).

Cells were seeded in 96-well plates at 8 x 10^4^ cells/well then incubated for 24 hours at 37°C, 5% CO_2_ and 95% humidity. Cells were then treated with the different fractions dissolved in DMSO (10 µg/mL). DMSO was used as negative control and doxorubicin as positive control. Three independent experiments were carried out. The plates were incubated for 72 hours. After treatment, the media were withdrawn and 20 µL MTT at 5 mg/mL PBS was added to each well. Plates were incubated for four hours, then 150 µL DMSO was added per well to dissolve the formazan crystals. Absorbance was read at 570nm using LeDetect microplate reader (Labexim, EU). Assuming 100% viability in control cells, inhibition was calculated using the formula:

$\mathrm{\%inhibition}=100\left(1-\frac{\mathrm{absorbance of cells treated with extract}}{\mathrm{absorbance of cells treated with DMSO}}\right)$

IC_50_ was determined using plots of percentages inhibition against concentrations with non-linear regression analysis using Graphpad Prism 6.0 software (San Diego, CA). 

*Cell selectivity of the cytotoxic HPLC isolate*

A promising anticancer compound should be specifically toxic against cancer cells but with minimal or no effect on non-cancer cell models. Recent researches are primarily directed towards discovering potent anticancer agents that are non-toxic to normal cells. The selectivity index (SI) value shows the selective toxicity of the sample to the cell lines tested. SI value ≥ 2 indicates promising selectivity against cancer cell lines (de Oliveira et al., 2015).

The cytotoxicity of HPLC7 fraction against HK-2 cells was determined as previously described. The SI of HPLC7 was calculated using the formula: 

SI=mean IC50of HK-2IC50of HCT 116

*Apoptotic effect of HPLC7 on HCT116*

HPLC7 demonstrated cytotoxicity and good selectivity against HCT116 hence, its apoptotic effect was investigated. 

*Documentation of cellular morphology*

The morphological features of cells treated with HPLC7 were observed through light microscopy. Apoptotic morphologies noted included cell shrinkage, nuclear condensation, cell rounding and detachment from substratum, membrane blebbing and formation of apoptotic bodies. Three independent trials in triplicates were performed. Five micrographs per replicate were captured after the experiment. 

*Analysis of mitochondrial membrane depolarization by JC-1 assay*

Since the intrinsic apoptosis pathway is characterized by decreased mitochondrial membrane potential, the effect of HPLC7 on mitochondrial membrane potential was investigated by JC-1 Assay (Invitrogen, CA, USA). Mitochondrial membrane depolarization, an early apoptotic event (Suzuki-Karasaki et al., 2013), was monitored after 24-hour treatment. HCT116 cells were seeded in a 96 well plate and were treated with DMSO and HPLC7 at IC_50 _(1.13 µg/mL) and IC_80_ (3.38 µg/mL) for 24 hours. Cells were treated with the positive control, carbonyl cyanide 3-chlorophenylhydrazone (CCCP) for 5 min prior to JC-1 staining. Cells were stained with JC-1 according to the manufacturer’s instruction with some modifications. Briefly, after exposure to treatments, JC-1 solution was added to the wells to a final concentration of 2μM followed by 30 min incubation. Cells were washed with PBS and fluorescence was measured using Varioskan LUX Multimode Microplate Reader (Thermo Fisher Scientific). Fluorescence was detected at Ex/Em 535/595nm for red and 485/535nm for green. Reduction in red to green fluorescence ratio is indicative of mitochondrial membrane depolarization. Three independent experiments were done with triplicates per experiment. Three representative micrographs per replicate were taken using a fluorescence microscope. 

*Detection of apoptosis by Caspase 3/7 assay*

Assessment of caspase 3/7 activation is essential in determining the execution of apoptosis in cells treated with a cytotoxic agent. CellEvent Caspase 3/7 ReadyProbes™ Reagent (Invitrogen, CA, USA), a fluorometric assay that allows monitoring of caspase 3/7 activation in treated cells over time, was used to determine if HPLC7 promotes apoptosis by activating caspase 3/7. Cells seeded in a 96-well plate were treated with HPLC7 (IC_50_ and IC_80_) after 24 hours. CellEvent Caspase 3/7 ReadyProbes™ reagent was added to each well to a final concentration of 2 µM. Plates were incubated and progression of apoptosis was checked every 24 hours. After 72 hours, cell nuclei were counterstained with Hoechst 33342 to a final concentration of 1 µg/mL (Invitrogen, CA, USA) and viewed using FITC/Alexa Fluor™ 488 filter settings in a fluorescence microscope at 400X magnification. Three independent experiments were performed in triplicates. Five representative micrographs were taken per replicate. The number of caspase positive cells were counted and the percentage of apoptotic cells was computed using the formula: 

$\mathrm{\%apoptotic cells}=100\frac{\mathrm{number of caspase}\frac{3}{7}\mathrm{positive cells}}{\mathrm{total number of cells counted}}$

*Detection of apoptosis by TUNEL assay*

DNA breaks in apoptotic cells treated with HPLC7 was determined using terminal deoxynucleotidyl transferase (TdT) mediated-16-deoxyyuridine triphosphate (dUTP) Nick-End Labelling (TUNEL) to visualize DNA fragmentation. DeadEnd™ fluorometric TUNEL system (Promega, WI, USA) was used according to the manufacturer’s protocol with some modification. HCT116 cells were seeded in 96-well plates and treated with the cytotoxic HPLC7 (IC_50_, and IC_80_) for 72 hours. Cells were harvested using trypsin, fixed in 4% formaldehyde, permeabilized with 0.2% Triton X-100 and labeled with TdT reaction mix. Hoechst 33342 was used to counterstain the nuclei. Stained cells were viewed using fluorescence microscope at 400X magnification. Three independent experiments in triplicates were performed. Five representative images per replicate were taken. The percentage of apoptotic cells was computed using the formula: 

$\mathrm{\%apoptotic cells}=100\left(\frac{\mathrm{number of TUNEL positive cells}}{\mathrm{total number of cells counted}}\right)$

*Statistical analysis*

The IC_50_ values are presented as means ± standard deviation (SD) of three independent experiments. Statistical differences were determined by one-way ANOVA followed by Tukey’s multiple comparison test or by the non-parametric Kruskal-Wallis test with a post-hoc Dunn’s test. All statistical analyses were performed using Graph Pad Prism 6.0. Differences were considered significant at P<0.05.

## Results

*Bioassay-guided purification of A. loheri extract*

The methanolic crude extract of *A. loheri* leaves was subjected to a bioassay-guided fractionation and cytotoxic activity of the fractions was evaluated on HCT116 by MTT assay. The cytotoxic HPLC isolate, a white amorphous powder, obtained after fractionation was determined as aglaforbesin derivative (AFD) by NMR analyses.

**Figure 1:**
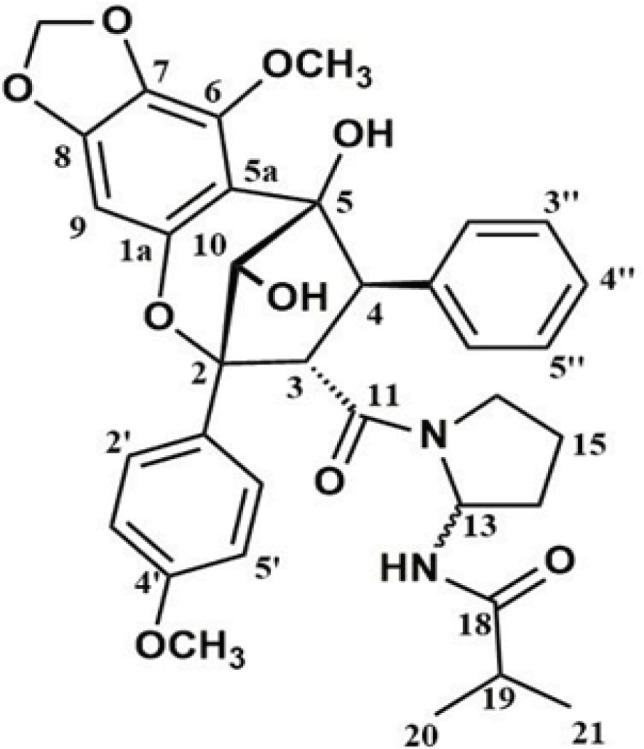
Structure of HPLC7, Aglaforbesin Derivative, AFD

The ^1^H NMR spectrum of HPLC7 isolate indicated the presence of two methyl signals at δ 0.85 (d, *J* = 6.6 Hz), and 0.90 (d, *J* = 6.6 Hz), two methoxy signals at δ 3.66 (s), and 3.78 (s), and two protons of dioxolo methylene at δ 5.92 (d, *J* = 1.8 Hz), and 6.18 (d, *J* = 1.8 Hz). Aromatic protons signals at δ 8.00 (m), 7.20 (m), 6.97 (m), 6.95 (m), 6.84 (m), and methylene and methine protons at δ 6.18 (d, *J* = 1.8 Hz), 5.92 (d, *J* = 1.8 Hz), 4.99 (m), 4.56 (d, *J* = 10.8 Hz), 3.93 (s), 3.61 (d, *J* = 1.8 Hz), 3.30 (m), 3.15 (m), 2.15 (m), 1.80 (m), 1.40 (m), and 1.25 (m) were also observed. The ^13^C NMR spectrum showed 31 carbon signals including two for methoxy carbons and two for amide carbons at δ 58.0, 55.5, 170.5, and 174.6, respectively. HSQC shows correlation between the benzodioxolo methylene protons at δ 5.92 (d, *J* = 1.8 Hz) and 6.18 (d, *J* = 1.8 Hz) and oxygenated carbon at δ 105.4. The oxymethine proton at δ 3.93 (s) is also directly bonded to the oxygenated carbon at δ 85.9 based on HSQC. Spectral data were carefully compared with previously published values for compounds isolated from *Aglaia* species giving a good match with the aglaforbesin derivative (Figure 1) previously isolated from the twigs of *Aglaia* *oligophylla* by Dreyer et al. (2001). The presence of piriferine-like substituent attached to C-3, and the aromatic substituents attached to C-4, and C-2 are supported by 1D and 2D NMR data.

*Cytotoxicity of A. loheri extracts*

The study revealed the plant to be a source of cytotoxic agents against cancer cells as demonstrated by the abundance of active fractions (Table 1). AFD isolated in the present study exhibited strong cytotoxicity against HCT116 with IC_50_ value of 1.13 ± 0.07 µg/mL but low toxicity to the normal HK-2 cells with IC_50_ value of 6.81 ± 1.8 µg/mL. The preliminary selectivity testing demonstrated in a high selectivity index of 6.04 against HCT116 (Supplementary Figure 1), increasing its potential for development as anticancer drug lead. AFD also demonstrated strong cytotoxicity but less selectivity against breast cancer (MCF7) and lung cancer (A549) cells (Supplementary Table1, Supplementary Figure 1). This is the first study to report on the potent cytotoxic activity of AFD against cancer cell lines. Previous investigation on AFD reported no bioactivity when tested against the larvae of *Spodoptera littoralis* (Dreyer et al., 2001). 

*AFD-induced apoptosis in HCT116 cells*

**Figure 2:**
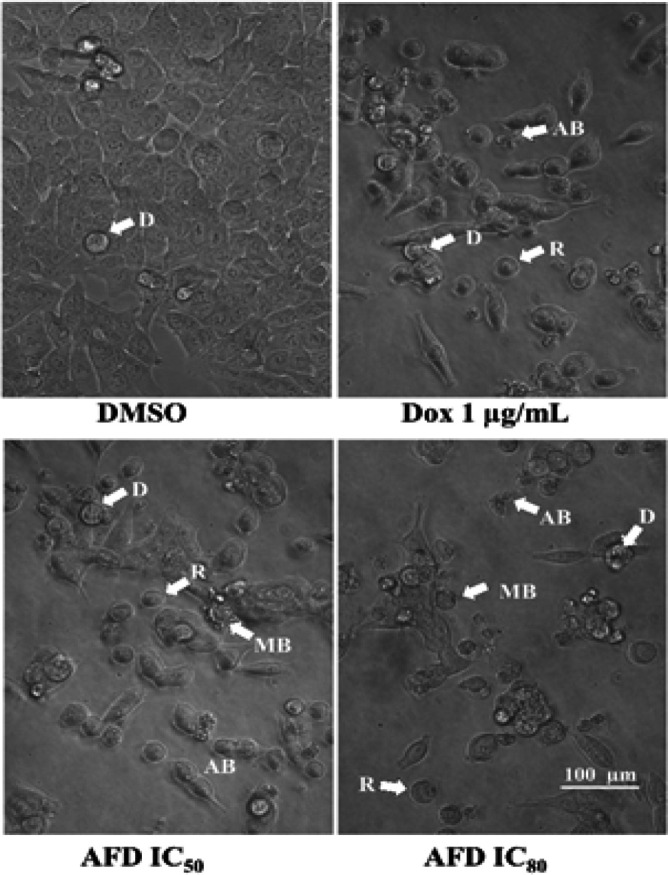
HCT116 Cells Treated with AFD for 72 hours Demonstrated Apoptotic Morphologies: detachment from substratum (D), rounding of cells (R), membrane blebbing (MB) and formation of apoptotic bodies (AB). Images are representative of three trials and were captured under bright field microscope

The effect of AFD on HCT116 cell morphology was evaluated after 72 hours treatment to determine apoptotic features. Figure 2 shows that cells treated with AFD exhibited characteristic features of apoptosis such as rounding and detachment from the substratum of culture plate, membrane blebbing and formation of apoptotic bodies also observed in other studies (Kroemer et al., 2009; Zhang et al., 2018). Hence, biochemical markers of apoptosis were investigated including mitochondrial membrane depolarization, caspase 3/7 activation, and DNA fragmentation.

**Figure 3:**
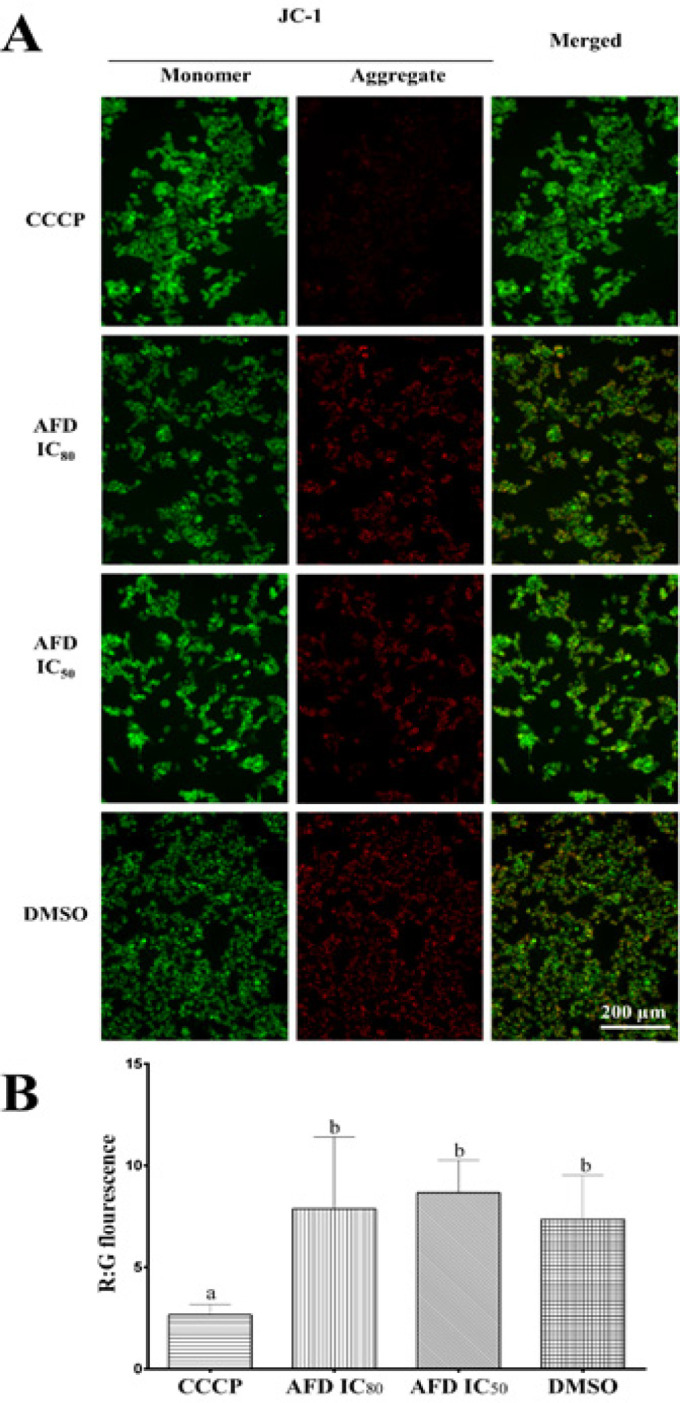
JC-1 Staining Showed that AFD Did not Induce Mitochondrial Membrane Depolarization in HCT116 Cells after 24 Hours Treatment. (A) Representative fluorescence images and (B) bar graph showing means ± SD of red to green fluorescence ratio showed no significant difference with set ups treated only with DMSO. Positive control cells were treated with carbonyl cyanide 3-chlorophenylhydrazone (CCCP). Means with different letters are significantly different (P<0.05).

To determine the effect of AFD treatment on mitochondrial membrane potential of HCT116 cells, JC-1 assay was used. Micrographs of AFD-treated cells showed no visible difference in red and green fluorescence from that of DMSO-treated cells (Figure 3A). The computed red to green fluorescence ratio of cells treated with AFD showed no significant difference from that of DMSO (Figure 3B). This shows that AFD did not induce mitochondrial membrane depolarization in HCT116 cells after 24 hours. 

**Figure 4:**
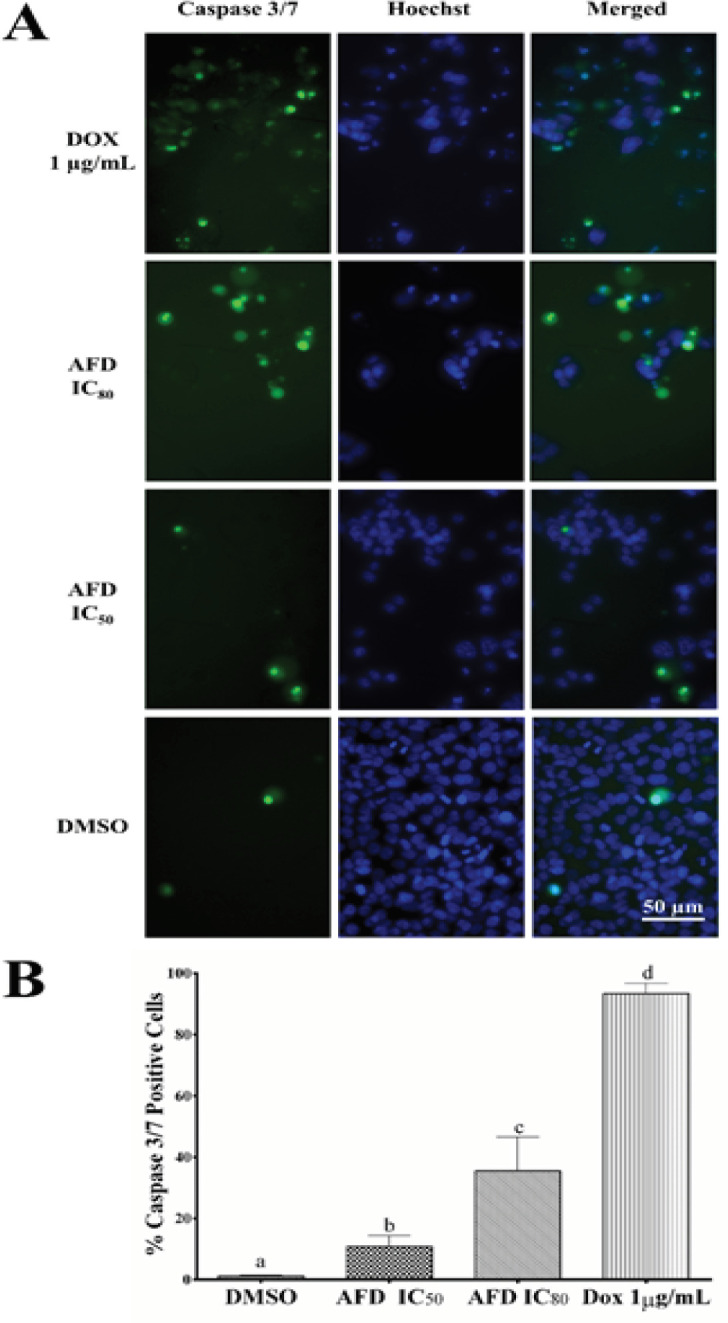
AFD Induced Apoptosis in HCT116 Cells Characterized by Increased Percentage of Caspase 3/7 Positive Cells. (A) Pictomicrographs of cells stained with CellEvent Caspase 3/7 ReadyProbes™ Reagent after 72 hours treatment with doxorubicin (1 µg/mL), AFD IC_80_, AFD IC_50_, and DMSO. Nuclei were counterstained with Hoechst 33342. (B) Bars represent mean % Caspase 3/7 positive cells from three trials with three replicates ± SD. Means with different letters are significantly different (P<0.05)

To further investigate apoptosis as the mode of cell death induced by AFD, caspase 3/7 activation was visualized by fluorescence microscopy. Representative micrographs and comparison of caspase 3/7 activation are shown in Figure 4A. Treatment of HCT116 with AFD IC_50_ for 72 hours showed caspase 3/7 activation in about 10% of cells while treatment with AFD IC_80_ showed increased caspase 3/7 activation to 35% (Figure 4B). However, caspase 3/7 activity of doxorubicin (93%) was significantly higher compared to DMSO and AFD IC_50 _treatments.

**Figure 5:**
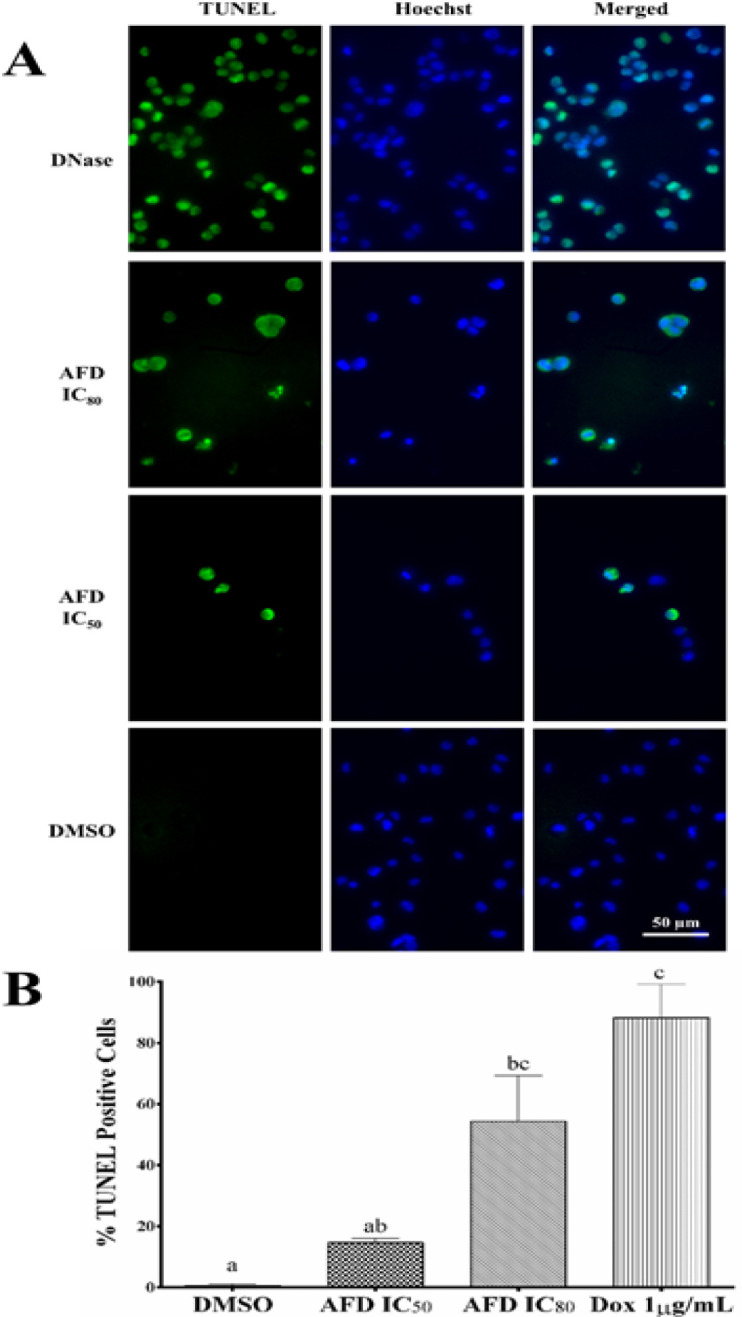
AFD Induced Apoptosis in HCT116 Cells Characterized by DNA Fragmentation Visualized after TUNEL Assay. (A) Pictomicrographs of cells after 72 hours treatment with AFD IC_80_, AFD IC_50_, and DMSO. DNase was used as positive control. Nuclei were counterstained with Hoechst 33342. (B) Bars represent mean % TUNEL positive cells from three trials with three replicates ± SD. Means with different letters are significantly different (P<0.05).

Although activation of caspases is suggestive of apoptosis, the presence of active caspases is not sufficient to define apoptosis (Kroemer et al., 2009). Hence, TUNEL assay was conducted which subsequently confirmed the apoptosis-inducing activity of AFD evidenced by DNA fragmentation. Activation of caspases, especially caspase 3, is known to induce DNA fragmentation and that leads to cell death (Kitazumi and Tsukahara 2011; Slee et al. 2001). Additionally, caspase 3 is essential to membrane blebbing by activating Rho-activated serine/threonine kinase ROCK1 that promotes the movement of DNA fragments to the bleb and the formation of apoptotic bodies (Zhang et al., 2018). Representative micrographs in Figure 5A showing intense green fluorescence of nuclei, were clearly detected in cells treated with AFD and DNase (positive control) but not in cells treated only with DMSO. Figure 5B showed occurrence of apoptotic DNA fragmentation in HCT116 cells treated with AFD IC_80_ (54.35%) that exhibit no significant difference from that of DNase (88.23%). However, treatment with AFD IC_50_ (14.61%) showed no significant difference from that of DMSO (0.62%). 

**Table 1:** Cytotoxicity* of *A. loheri *Fractions as Tested against Human Cancer Cells HCT116 and Normal Cells HK-2 for 72 h

Extract/Fractions	HCT116	HK-2
**Methanolic Crude**	1.83 ± 0.28	
Partitions:		
Hexane	1.77 ± 0.18	
**Ethyl Acetate**	0.57 ± 0.16	
Aqueous	>100	
GCC Fractions:		
23	0.19 ± 0.13	
24	0.47 ± 0.02	
**26**	3.96 ± 0.99	
28	1.99 ± 0.92	
29	0.69 ± 0.28	
30	0.15 ± 0.02	
31	0.13 ± 0.02	
32	0.39 ± 0.17	
33	0.2 ± 0.14	
34	0.51 ± 0.17	
35	3.13 ± 0.84	
FC Fractions:		
FC9	5.06 ± 0.52	
**FC10**	4.97 ± 0.52	
SEC Fractions:		
**SEC7**	6.34 ± 0.56	
SEC8	6.08 ± 4.68	
HPLC:		
** HPLC7/AFD**	1.13 ± 0.02	6.81 ± 1.80
Doxorubicin	0.14 ± 0.02	0.67 ± 0.43

*IC_50_ (µg/mL): concentration that inhibits cell viability by 50%; Fractions selected in each fractionation process are in bold text. Doxorubicin was used as positive control. Values represent means ± SD, n = 3.

## Discussion

The genus *Aglaia* has received considerable attention in the past decades after the isolation of rocaglamide and their related compounds which demonstrated potential significance in agro-chemistry and pharmacology (Ebada et al., 2011). In the present study, the bioassay-guided fractionation of *A. loheri* leaf extracts revealed the potential of the species as source of cytotoxic agents against human colorectal cancer as demonstrated by the abundance of cytotoxic fractions against HCT116. Most importantly, this investigation lead to the isolation of the selectively cytotoxic agent which was structurally elucidated as aglaforbesin derivative. 

AFD demonstrated good cytotoxic activity similar to previously discovered aglaforbesin compounds that demonstrate promising anticancer activity. Foveoglin A isolated from leaves of *Acrosticta foveolata* displayed cytotoxicity against Lu1 (ED50=1.8 µM), LNCaP (ED_50_=1.4 µM) and MCF7 (ED_50_=1.8 µM) (Salim et al., 2007). Perviridisin B from *Aglaia perviridis* exhibited selective cytotoxicity to HT29 cells (ED_50_=0.46 µM) and inactive against normal colon cell line CCD112CoNl (ED_50_>50 µM) (Pan et al., 2013). Additionally, aglaodoratin D from A. odorata demonstrated significant cytotoxicity against human osteosarcoma carcinoma MG-63 (An et al., 2015). Prior to the isolation of foveoglin A, it was thought that only cyclopenta[b]benzofuran type of flavaglines are cytotoxic against cancer cell lines and that cyclopenta[b]benzopyran representatives are inactive (Salim et al., 2007). They attributed this cytotoxic activity to the type of amide moiety, the positions of substituents at C-3 and C-4 and the orientation of the OH-10 (Salim et al., 2007). AFD contains a piriferine-like substituent attached to C-3 and a phenyl ring attached to C-4 (Dreyer et al., 2001). As determined in this study, AFD is an addition to the list of cyclopenta[b]benzopyran type of flavaglines that exhibit cytotoxic activity against cancer cell lines.

Having established the selective cytotoxicity of AFD towards HCT116, the investigation then focused on the mode of cell death it induced. Microscopic examination of the cells treated with AFD revealed the different morphological features of apoptosis. This led to investigate the biochemical markers of apoptosis: mitochondrial membrane depolarization, caspase 3/7 activation, and DNA fragmentation. Results showed that AFD-induced apoptosis in HCT116 cells involved caspase 3/7 activation and DNA fragmentation. Activation of caspases by other Aglaia-derived compounds have been reported previously. Rocaglamide and silvestrol, are known to induce caspase 3 activation in leukemia cell and MDA-MB-435 melanoma cells, respectively (Chen et al., 2016; Zhu et al., 2007). Both compounds are also known to induce intrinsic apoptosis though mitochondrial membrane depolarization in acute myelogenous leukemia cells, prostate cancer cells and chronic lymphocytic leukemia cells (Lucas et al., 2009; Callahan et al., 2014; Hwang et al., 2004). However, AFD failed to induce mitochondrial membrane depolarization at 24 hours indicating that the cytotoxic activity of AFD may have occurred later or apoptosis occurred independent of mitochondrial membrane depolarization. These differences in bioactivity may be attributed to structural difference of the compounds. Both rocaglamide and silvestrol contain the cyclopenta[b]benzofuran framework while AFD contains a cyclopenta[bc]benzopyran framework.

In conclusion, the present study demonstrated favorable selective cytotoxicity against cancer cells by aglaforbesin derivative isolated from A. loheri. This cytotoxicity may be related to the activation of the apoptotic pathway. This warrants further exploration into the compound as potential anticancer agent, especially against colorectal cancer. The mechanism of action of aglaforbesin derivative leading to toxicity and apoptosis should be investigated further especially since this differs from the action of other known rocaglamides from Aglaia.
